# A novel transcranial photobiomodulation device to address motor signs of Parkinson's disease: a parallel randomised feasibility study

**DOI:** 10.1016/j.eclinm.2023.102338

**Published:** 2023-12-01

**Authors:** Geoffrey Herkes, Claire McGee, Ann Liebert, Brian Bicknell, Vivian Isaac, Hosen Kiat, Craig S. McLachlan

**Affiliations:** a Department of Neurology, Sydney Adventist Hospital, Wahroonga, NSW, 2076, Australia; b Faculty of Health Sciences, Torrens University Australia, Sydney, NSW, 2000, Australia; c Sydney Adventist Hospital, Wahroonga, NSW, 2076, Australia; d School of Medical Sciences, University of Sydney, Camperdown, NSW, 2050, Australia; e NICM Health Research Institute, University of Western Sydney, Westmead, NSW, 2145, Australia; f School of Allied Health, Exercise & Sports Sciences, Charles Sturt University, Albury, NSW, 2640, Australia; g Faculty of Medicine, Human and Health Sciences, Macquarie University, Sydney, NSW, 2109, Australia; h College of Health and Medicine, Australian National University, Canberra, ACT, 2601, Australia; i Cardiac Health Institute, Sydney, NSW, 2010, Australia; j Centre for Healthy Futures, Torrens University Australia, Sydney, NSW, 2000, Australia

**Keywords:** Parkinson's disease, Photobiomodulation, Transcranial, Movement disorders, UPDRS

## Abstract

**Background:**

Parkinson's disease is a progressive neurological disease with limited treatment options. Animal models and a proof-of-concept case series have suggested that photobiomodulation may be an effective adjunct treatment for the symptoms of Parkinson's disease. The aim was to determine the safety and feasibility of transcranial photobiomodulation (tPBM) to reduce the motor signs of Parkinson's disease.

**Methods:**

In this double-blind, randomised, sham-controlled feasibility trial, patients (aged 59–85 years) with idiopathic Parkinson's disease were treated with a tPBM helmet for 12 weeks (72 treatments with either active or sham therapy; stage 1). Treatment was delivered in the participants' homes, monitored by internet video conferencing (Zoom). Stage 1 was followed by 12 weeks of no treatment for those on active therapy (active-to-no-treatment group), and 12 weeks of active treatment for those on sham (sham-to-active group), for participants who chose to continue (stage 2). The active helmet device delivered red and infrared light to the head for 24 min, 6 days per week. The primary endpoints were safety and motor signs, as assessed by a modified Movement Disorders Society revision of the Unified Parkinson's Disease Rating Scale Part III (MDS-UPDRS-III)-motor scale. This trial is registered with ANZCTR, ACTRN 12621001722886.

**Findings:**

Between Dec 6, 2021, and Aug 12, 2022, 20 participants were randomly allocated to each of the two groups (10 females plus 10 males per group). All participants in the active group and 18 in the sham group completed 12 weeks of treatment. 14 participants in the sham group chose to continue to active treatment and 12 completed the full 12 weeks of active treatment. Treatment was well tolerated and feasible to deliver, with only minor, temporary adverse events. Of the nine suspected adverse events that were identified, two minor reactions may have been attributable to the device in the sham-to-active group during the active treatment weeks of the trial. One participant experienced temporary leg weakness. A second participant reported decreased fine motor function in the right hand. Both participants continued the trial. The mean modified MDS-UPDRS-III scores for the sham-to-active group at baseline, after 12 weeks of sham treatment, and after 12 weeks of active treatment were 26.8 (sd 14.6), 20.4 (sd 12.8), and 12.2 (sd 8.9), respectively, and for the active-to-no-treatment group these values were 21.3 (sd 9.4), 16.5 (sd 9.4), and 15.3 (sd 10.8), respectively. There was no significant difference between groups at any assessment point. The mean difference between groups at baseline was 5.5 (95% confidence interval (CI) −2.4 to 13.4), after stage 1 was 3.9 (95% CI −3.5 to 11.3 and after stage 2 was −3.1 (95% CI 2.7 to −10.6).

**Interpretation:**

Our findings add to the evidence base to suggest that tPBM is a safe, tolerable, and feasible non-pharmaceutical adjunct therapy for Parkinson's disease. While future work is needed our results lay the foundations for an adequately powered randomised placebo-controlled clinical trial.

**Funding:**

SYMBYX Pty Ltd.

**Research in context**

**Evidence before this study**

Parkinson's disease is a progressive neurodegenerative disease. The treatment options include both medications to replace dopamine in the brain to manage some symptoms and invasive deep brain stimulation or ultrasound ablation to control tremors. There is scope for additional complementary therapies to further improve Parkinson's disease outcomes. Photobiomodulation (PBM) has been shown in animal models to alleviate the clinical signs of Parkinson's disease and to be neuroprotective and neuro-regenerative in toxin induced rodent models of Parkinson's disease. There have also been a number of small proof-of-concept case studies that have suggested some benefit of using an extracranial helmet to deliver transcranial PBM for Parkinson's disease symptoms. However, it is still unclear whether the use of PBM on the head alone could offer benefits. We aimed to investigate the safety and feasibility of a novel transcranial light emitting diode helmet as an adjunctive therapy for Parkinson's disease.

**Added value of this study**

In this parallel randomised feasibility trial, PBM was delivered transcranially to the head of patients with Parkinson's disease, and the safety and feasibility of the therapy to modify motor signs of Parkinson's disease was assessed. There was no significant difference in the mean scores of the modified Movement Disorders Society revision of the Unified Parkinson's Disease Rating Scale Part III (MDS-UPDRS-III) between active and sham treatments after 12 weeks of treatment. However, there was added improvement in the modified MDS-UPDRS-III for the majority of participants when sham treatment was replaced with active treatment, pointing to the need for a larger, adequately powered study.

**Implications of all the available evidence**

Our findings add to the evidence base to suggest that transcranial PBM is both a safe and feasible adjunct treatment for Parkinson's disease. The effect of transcranial PBM in the observed changes in motor signs of Parkinson's disease, combined with evidence from previous clinical trials and animal studies, has potential implications for the effective treatment of Parkinson's disease, using transcranial PBM as an adjunctive treatment combined with traditional dopamine medications. Further investigation is needed. This study lays the foundations for an adequately powered randomised placebo-controlled clinical trial to be performed in the future.

## Introduction

Parkinson's disease has a complex neuropathology, with much patient heterogeneity due to the progressive loss of dopaminergic neurons in the substantia nigra, producing the classic changes in motor function including bradykinesia, tremor, rigidity, and postural instability that invariably progress over time.^1^ While there are several treatment options available to manage the motor signs and symptoms of Parkinson's disease, including pharmaceutical options such as dopamine replacement and dopamine agonists as well as therapeutic devices, such as deep brain stimulation and non-invasive devices,^2^ treatment remains a challenge with further options needed.^3^

Photobiomodulation (PBM) is a therapy that involves the use of non-thermal red and near-infrared light to stimulate cellular function and promote tissue repair and regeneration.^4^ The therapeutic effects of PBM include pain reduction,^5^ improved circulation,^6^ wound healing,^7^ and reduced inflammation including neuroinflammation.^8^ Recently, PBM has been included in the National Institute for Health and Care Excellence (NICE) and the Multinational Association of Supportive Cancer Care (MASCC) guidelines to treat oral mucositis resulting from cancer therapies.^9^

The use of transcranial PBM (tPBM) has been explored in translational models of neurodegenerative and neurological diseases such as Alzheimer's disease,^10^^,^^11^ traumatic brain injury,^12^ stroke,^13^ and anxiety/depression.^14^^,^^15^ While its use for Parkinson's disease has been limited,^16^ its potential as a therapy has been recognised,^17^^,^^18^ and a recent proof-of-concept study has shown that tPBM combined with abdominal PBM, can control motor symptoms of Parkinson's disease for three years with continued treatment.^19^^,^^20^ It is, however, uncertain whether benefits could be gained by use of PBM on the head alone.

The aim of this study was to investigate the safety and feasibility of a novel transcranial light emitting diode (LED) helmet as an adjunctive therapy for Parkinson's disease.

## Methods

### Study design and participants

This trial was a parallel, randomised feasibility trial, conducted for 12 weeks, with the option for those receiving the sham intervention to complete an additional 12 weeks with active treatment. Due to coronavirus disease 2019 (COVID-19) restrictions, the original trial design was modified. The study was run entirely remotely in participants' homes with participants in Australia. Participant enrolment, training in use of devices, and participant assessments were conducted via internet video link (Zoom). Participants and care givers were contacted at the minimum every two weeks via Zoom, email, or phone to monitor safety and compliance, and to answer any questions or concerns regarding fitting of the helmet device, its usage, or side-effects. Compliance and side-effects were also monitored by the care giver. The trial protocol has been previously described.^21^

Participants with diagnosed Parkinson's disease were recruited from TV advertisements and selected according to inclusion/exclusion criteria, updated to accommodate at-home treatment and assessment (Supplementary Table S1) from a pool of over 300 people with Parkinson's disease who contacted the researchers. Sex was self-reported as male or female.

The trial was approved by the Sydney Adventist Health Human Ethics Research Committee, approval number (2019–032). All participants provided written informed consent to participate in this study. The trial was registered with Australian New Zealand Clinical Trial Registry (ANZCTR) a primary registry in the WHO Registry Network. Australian Clinical Trials Registry Number (ACTRN) 12621001722886.

### Randomisation and masking

Participants were randomly assigned to either the sham group or the active group using a computer random number generator, by a researcher not involved in participant contact, training, assessment, or data analysis. Participants were informed by registered mail of their group allocation and enrolled over Zoom by a researcher who was also an assessor but blinded to the group allocation (CM). The active tPBM helmet and the sham helmet looked and operated identically, except that the sham helmet device produced no light. Participants in the sham group were told that the helmet produced infrared (IR) light that could not be seen. Participants received helmet devices by post and were instructed on how to fit and operate, as well as the treatment regimen by a single researcher who was the trial technical advisor and the only researcher not blinded to the treatments. All other researchers (assessors, data analysts) were blinded to group allocation, including when participants were unblinded after the first 12 weeks of treatment. The success of the masking was assessed by the technical advisor during regular Zoom calls.

### Treatment

**Fig. 1:**
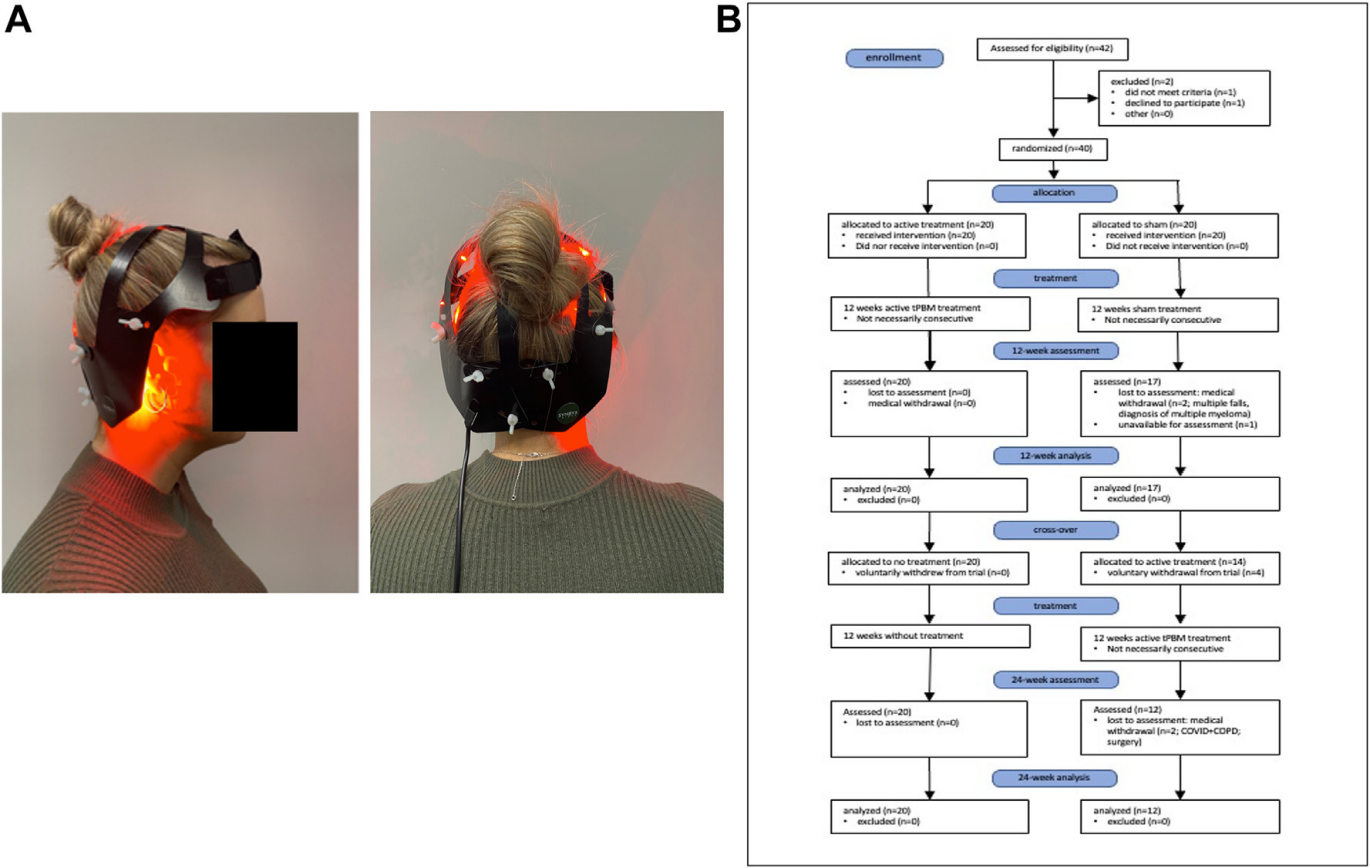
A: tPBM “Neuro” helmet device, lateral and posterior views (the sham device was identical but with no light emitted); B: CONSORT flow diagram.

The active treatment was a purpose-designed tPBM helmet “Neuro” manufactured by SYMBYX Pty Ltd, Sydney, Australia (Fig. 1A) with 40 LED diodes (20 red - 635 nm + 20 infrared - 810 nm) in 20 locations. Average optical power for the 810 nm LED was 52 mW and for the 635 nm LED was 27 mW. Treatment consisted of 12 min of red followed by 12 min of infrared irradiation, giving a total energy of 748.8J (IR) and 388.8 (red), delivered six days per week for 12 weeks (72 total treatments). The sham device appeared identical but delivered no light. Daily timing of the treatments was left up to individual participant preference. Participants were instructed to apply tPBM therapy consistently at the same time of day if possible. This was monitored by the trial technical advisor who reported almost universal consistency in tPBM therapy timing by participants.

In stage 1 of the study, the sham group was treated with the sham tPBM helmet and the active group with the active tPBM helmet. After 72 treatments the participants were unblinded and in stage 2 of the study, the sham group was offered the opportunity of 12 weeks of active treatment (sham-to-active group) and the active group received no treatment.

Some participants reported technical problems (mostly battery connection issues) with the helmet device that could not be rectified with a Zoom meeting. This resulted in return of the device to the manufacturer, repair and redispatch to the participant. This deviation from the preferred protocol resulted in the 12-week treatment period extending beyond the original end of treatment date allocated in the protocol for some participants in order to complete the 72 treatments. Participants were instructed to make no change to their medication, exercise regimen or diet.

### Outcomes

**Fig. 2:**
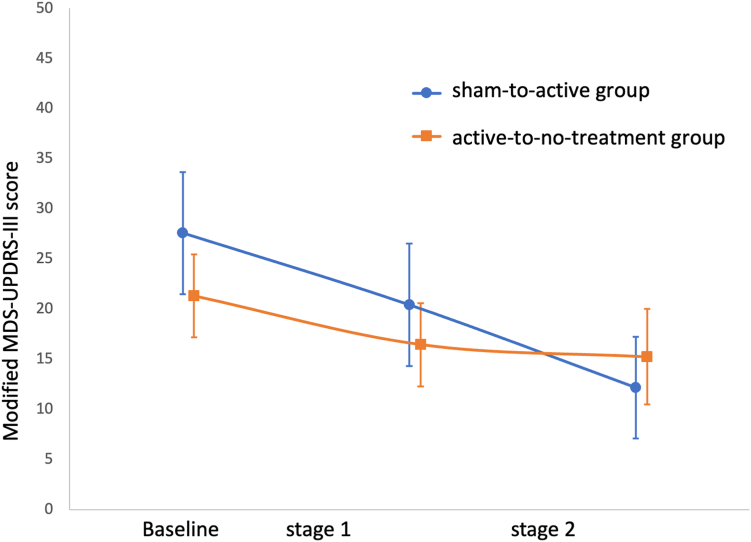
Change in mean modified MDS-UPDRS-III. sham-to-active group—sham for 12 weeks of treatment followed by active tPBM for 12 weeks of treatment; active-to-no-treatment group—active tPBM for 12 weeks of treatment followed by no treatment for 12 weeks. Error bars are 95% confidence intervals.

The primary outcomes were safety and motor outcomes. Safety was assessed by monitoring of the participants by the trial technical advisor during weekly and second weekly virtual meetings. Any concerns regarding the safety of the devices, side effects of treatment or other adverse events brought to the attention of the trial technical advisor, whether attributable to the treatment or not, were recorded as a suspected adverse event (SAE) and reviewed by an SAE committee who were blinded to the participant groups (Fig. 2).

Motor outcomes were assessed with a modified Movement Disorders Society revision of the Unified Parkinson's Disease Rating Scale Part III (MDS-UPDRS-III) (motor) assessment. The modified MDS-UPDRS-III consisted of removing one item (postural instability) that was considered to be unsafe when conducted remotely and a second item (rigidity) that could not be reliably assessed using internet video conferencing. This version of the MDS-UPDRS-III has been previously validated for remote delivery.^22^ Secondary outcome measures are the subject of a separate report.

Outcomes were assessed at baseline, after 12 weeks of treatment (stage 1), and after a further 12 weeks of either treatment or no treatment (stage 2) by assessors (GH, CM, plus 2 others) who were trained to administer the modified MDS-UPDRS-III remotely by a neurologist (GH). All assessors were unaware of treatment assignment throughout the trial.

### Statistical analysis

The researcher/statistician (VI) who analysed the data was blinded to the groups. As a feasibility study, there was no previous data to determine the power of the study. Statistical analysis was performed using SPSS version 29.0.0.0. Data was checked for errors and assumptions for statistical analysis, including normality of the data, by visual inspection of the equality of variance of the residuals. Independent-sample t-tests were conducted to investigate the difference in modified MDS-UPDRS-III scores between groups at baseline, after stage 1 and after stage 2. Data curation was monitored by an independent researcher following International Organization for Standardization (ISO) 14155 Medical Device Certification guidelines.

### Role of the funding source

The funder had no role in study design, data collection, data analysis, data interpretation, writing of the report or the decision of where to publish.

## Results

40 participants were enrolled in the trial (Table 1) and were included in the analysis. The trial ran from December 2021 to August 2022, with the first participant enrolled on December 6th, 2021, and the first four participants beginning treatment on December 23rd. The final participant completed the treatment with the active helmet on August 12th, 2022. The trial profile is shown in Fig. 1B. Compliance in stage 1 was excellent, with no withdrawals from the active group and two from the sham group due to unrelated medical issues (Table 2). There were six additional withdrawals from the sham-to-active group in stage 2, the active treatment part of the trial (two with unrelated medical conditions and four voluntary withdrawals). The number of participants who chose not to continue produced a disparity between the numbers in each group during stage 2 (20 vs 12), which has the potential to bias the results. Ten participants had disrupted treatment due to return and redispatch of the helmet device. In addition, 5 participants had disrupted treatment due to holiday, surgery, or minor reactions to the device. This disruption ranged from 3 days to 8 weeks due to COVID-19 and logistic related delays. All participants with disrupted treatment continued for the full 72 treatments and were included in the analysis. All participants with disrupted tPBM treatment showed an improvement in modified MDS-UPDRS-III scores over the 24 weeks of treatment despite these interruptions. There was no significant difference in age and sex distribution between groups (Table 1).

**Table 1:** Participant characteristics.

Participant	Sex	Age	Diagnosis (years)	Medication (L/S)	LdED
**Sham group**					
1	M	80	14	L	800 mg
4	F	71	5	L	200 mg
5	F	75	6	L	450 mg
6	M	69	5	L	700 mg
8	F	78	6	L	850 mg
12	F	61	3	L	100 mg
14	M	74	17	L,S	600 mg
15	M	64	5	L	400 mg
19	F	69	5	L	900 mg
20	M	73	6	L	600 mg
21	F	65	2	L	300 mg
23	M	66	6	L	400 mg
27	M	69	10	L	800 mg
29	F	75	5	L	200 mg
30	M	72	2	L	200 mg
32	M	71	1	L	NA
33	F	78	11	L	800 mg
35	M	70	1	L	NA
36	F	71	3	L	400 mg
38	F	66	3	L	300 mg
**Mean**	**10xF; 10xM**	**70.9**	**5.8**		
**Active group**					
2	M	79	7	L	600 mg
3	F	67	3	L	300 mg
7	M	77	5	L	450 mg
9	M	67	6	L	NA
10	M	72	3	L	700 mg
11	F	77	5	L	300 mg
13	M	79	10	S	100 mg
16	F	72	2	L	300 mg
17	F	76	4	L	300 mg
18	F	78	1	L	300 mg
22	M	69	5	L	400 mg
24	M	69	4	L	300 mg
25	F	72	1	nil	
26	M	61	1	L	300 mg
28	M	79	3	L	450 mg
31	M	75	5	L	400 mg
34	F	77	5	nil	
37	F	78	4	L	150 mg
39	F	73	3	S	200 mg
40	F	69	10	L	300 mg
**Mean**	**10xF 10xM**	**73.3**	**4.4**		
**Mean (both groups)**	**20xF 20xM**	**72.0**	**5.1**		

M = male, F = female, L = L-dopa, S=Silfol, LdED = L-dopa equivalent dose, NA = data not available, nil = not on anti-Parkinson's disease medication.

**Table 2:** MDS-UPDRS-III (modified) scores and changes, including side effects and reactions.

Sham-to-active participant	Baseline	After stage 1	Change baseline to after stage 1 (% change)	After stage 2	Change after stage 1 to after stage 2 (% change)	Change baseline to after stage 2 (% change)	Comments and SAEs
1	21	18	−3 (−14.3)	withdrew			voluntary withdrawal
4	19	10	−9 (−47.5)	8	−2 (−20.0)	−11 (−57.9)	SAE temporary dizziness during sham treatment—continued treatment
5	24	28	4 (16.7)	30	2 (7.1)	6 (25.0)	SAE leg weakness & dizziness active treatment—continued treatmentˆ
6	20	14	−6 (−30.0)	5	−9 (−64.3)	−15 (−75.0)	SAE temporary dizziness during sham treatment—continued treatment
8	18	5	−13 (−72.2)	withdrew			voluntary withdrawal
12	22	withdrew					SAE diagnosed with multiple myeloma—not device associated
14	36	23	−13 (−36.1)	withdrew			SAE medical withdrawal during active treatment (COVID-19 and COPD)
15	29	33	4 (13.8)	16	−17 (−51.5)	−13 (−44.8)	
19	34	24	−10 (−29.4)	12	−12 (−50.0)	−22 (−64.7)	SAE surgery; medication change during active treatment continued treatment
20	30	15	−15 (−50.0)	withdrew			voluntary withdrawal
21	15	6	−9 (−60.0)	6	0	−9 (−60.0)	
23	42	withdrew		withdrew			SAE multiple falls during sham treatment—not device associated
27	34	33	−1 (−2.9)	27	−6 (−18.2)	−7 (−20.6)	
29	17	30	13 (76.5)	withdrew			withdrawal after surgery during active treatment—not device associated
30	46	unavailable		11	–	−35 (−76.1)	unavailable for assessment at 12 weeks
32	9	5	−4 (−44.4)	5	0	−4 (−44.4)	
33	58	46	−12 (−20.7)	18	−28 (−60.9)	−40 (−69.0)	
35	15	5	−10 (−66.7)	3	−2 (−40.0)	−12 (−80.0)	SAE mild hand dystonia during active treatment—continued treatment^a^
36	53	39	−14 (−26.4)	withdrew			voluntary withdrawal
38	10	13	3 (30.0)	5	−8 (−61.5)	−5 (−50.0)	
**mean**	**26.8**	**20.4**	**−5.59 (**−**19.1)**	**12.2**	**−7.45 (**−**29.9)**	−13.9 (−51.5)	
**Active-to-no-treatment participant**	**Baseline**	**After stage 1**	**Change baseline to after stage 1 (% change)**	**After stage 2**	**Change after stage 1 to after stage 2 (% change)**	**Change baseline to after stage 2 (% change)**	**Comments and SAEs**

2	21	35	14 (66.8)	43	8 (22.8)	22 (104.7)	
3	8	4	−4 (−50.0)	3	−1 (−25.0)	−5 (−62.5)	
7	28	21	−7 (−25.0)	22	1 (4.7)	−6 (−21.4)	
9	43	18	−25 (−58.1)	16	−2 (−11.1)	−27 (−62.8)	SAE 2 minor car accidents during active treatment weeks—continued treatment
10	24	18	−6 (−25.0)	28	10 (55.6)	4 (16.7)	
11	19	12	−7 (−36.8)	12	0	−7 (−36.8)	
13	19	21	2 (10.5)	20	−1 (−4.8)	1 (5.3)	
16	16	9	−7 (−43.8)	4	−5 (−55.6)	−12 (−75.0)	
17	16	17	1 (6.3)	7	−10 (−58.8)	−9 (−56.3)	
18	26	21	−5 (−19.2)	24	3 (14.2)	−2 (−7.7)	
22	36	29	−7 (−19.4)	14	−15 (−51.7)	−22 (−61.1)	
24	8	2	−6 (−75.0)	4	2 (100.0)	−4 (−50.0)	
25	17	5	−12 (−70.6)	5	0	−12 (−70.5)	
26	9	3	−6 (−66.7)	4	1 (33.3)	−5 (−55.6)	
28	17	25	8 (47.1)	22	−3 (−12.0)	5 (29.4)	
31	21	11	−10 (−47.6)	5	−6 (−54.4)	−16 (−76.2)	
34	30	23	−7 (−23.2)	14	−9 (−39.1)	−16 (−53.3)	
37	26	23	−3 (−11.5)	18	−5 (−21.7)	−8 (−30.8)	
39	11	6	−5 (−45.5)	9	3 (50.0)	−2 (−18.2)	
40	32	26	−6 (−18.8)	31	5 (19.2)	−1 (−3.1)	
**mean**	**21.3**	**16.5**	**−4.85 (**−**25.3**)	**15.3**	**−1.3 (**−**7.7)**	−6.1 (−29.3)	

SAE = suspected adverse outcome.

a = potentially a reaction to the helmet device.

The mean modified MDS-UPDRS-III scores for the sham-to-active group at baseline, after stage 1 and after stage 2 were 26.8 (standard deviation (sd) 14.6), 20.4 (sd 12.8) and 12.2 (sd 8.9) respectively and for the active-to-no-treatment group were 21.3 (sd 9.4), 16.5 (sd 9.4) and 15.3 (sd 10.8) (Table 3). The mean modified MDS-UPDRS-III scores between groups were not significantly different at baseline, after stage 1, or after stage 2. There was individual variation in response to tPBM and sham with a number of participants showing an increase in modified MDS-UPDRS-III score.

**Table 3:** Mean MDS-UPDRS-III (modified) scores at baseline, after stage 1 and after stage 2.

	Sham-to-active		Active-to-no-treatment		Mean difference
	n	Mean (sd)	n	Mean (sd)	(95% CI)
Baseline	20	26.8 (14.6)	20	21.3 (9.4)	5.5 (−2.4 to 13.4)
After stage 1	17	20.4 (12.8)	20	16.5 (9.4)	3.9 (−3.5 to 11.3)
After stage 2	12	12.2 (8.9)	20	15.3 (10.8)	−3.1 (2.7 to −10.6)

sd = standard deviation; CI = confidence interval.

Of the nine SAEs that were identified during the trial (Table 2), only two minor reactions may have been attributable to the device in the sham-to-active group during the active treatment weeks of the trial. One participant (5) experienced leg weakness and, although advised to withdraw, continued treatment. The weakness subsided over the next two weeks. A second participant (35) reported decreased fine motor function in the right hand but also continued the trial. There were two reports of transient minor dizziness during sham helmet treatment and one during active tPBM treatment.

## Discussion

The findings suggest that tPBM is a safe and feasible treatment to address motor signs of Parkinson's disease. The safety of the tPBM treatment is consistent with other studies of tPBM, including a study that specifically examined side effects and reactions of tPBM.^23^ There was a small number of side effects that were minor and transient, being reversed after a few weeks or with treatment cessation. Compliance was excellent, although four participants after sham therapy declined to begin treatment with the active therapy.

While there was no significant difference between active and sham groups after stage 1, a further examination of the individual components of the modified MDS-UPDRS-III scores has revealed differences between active and sham, with responders to active treatment having significant improvement in five modified MDS-UPDRS-III sub-scores, compared to one sub-score for sham treatment.^24^ A placebo effect, due to being included in a clinical trial, may explain the positive response to the sham helmet at 12 weeks that was indistinguishable from active treatment. Placebo effects are known to influence Parkinson's disease trial treatments due to the release of dopamine associated with response to any intervention, including placebo.^25^

The overall improvement for many participants in the sham group when they progressed to active treatment suggests a positive signal to tPBM above placebo and points to the need to conduct a larger, appropriately powered, randomised crossover trial. In addition, while some participants in the active group declined after cessation of treatment, others continued to improve, suggesting some stability of the tPBM treatment, at least for some participants.

The exact mechanism of action of tPBM for the symptoms of Parkinson's disease is unclear. In animal models PBM has been shown to reduce neuroinflammatory astrocyte and microglial responses, reduce inflammatory cytokines, and attenuate reactive oxygen species.^8^ Interestingly, in animal Parkinson's disease models, tPBM has been shown to be neuroprotective.^26^ In humans, however, tPBM will not penetrate to the substantia nigra where dopaminergic loss occurs, which necessitates proposing alternate mechanisms.^27^ These might include enhancing glymphatic drainage from the brain^28^ and/or light stimulation of the vagus nerve and the putative endorestiform nucleus.^29^ Both mechanisms might occur due to the placement of the LEDs below the posterior base of the skull.

The clinical study had a number of limitations. First, as a feasibility trial, the study was under powered, especially when the number of dropouts from the sham-to-active group is considered. This would have the potential to bias the results and limit interpretation. The selection criteria for participants also potentially introduced bias, with the need for care givers, familiarity with technology and adequate space for web-based assessments. The trial ran during the COVID-19 pandemic, necessitating changes to the protocol and modification of the MDS-UPDRS-III to a version that had been previously validated. The trial was conducted entirely remotely, with no face-to-face interaction between participants and therapists or assessors, instead using the assistance of care givers. This allowed, however, for a real-world pragmatic study. Technical issues with some helmet devices resulted in some loss of treatment continuity. Nevertheless, even with treatment interruption, there was still an improvement in motor scores. Individual participant differences, such as circadian rhythms and hair colour that will absorb red and infrared light to different extents, may have affected the outcome of tPBM. It is also possible, although unlikely, that some participants in the sham group might have discovered that they had an inactive device by an internet search of similar helmet devices. However, feedback to the trial technical advisor from participants after they had been unblinded indicated an almost universal belief that the helmet had been active. Finally, numbers were modest and the 12-week treatment regimen was short for a progressive chronic disease, however the data was sufficient to determine safety of the tPBM helmet device and the feasibility of the treatment.

In conclusion, this study has shown that the transcranial PBM treatment is safe and is feasible to be delivered as a non-pharmaceutical adjunct therapy for Parkinson's disease. The trial results support and extend earlier published proof-of-concepts studies,^19^^,^^20^ and provides valuble information for a larger, suitably powered randomised placebo-controlled trial.

## Contributors

CM wrote the first draft of the manuscript; BB, AL, CSM, GH and HK contributed content to the drafts; VI did the statistical analysis; all authors had full access to the data; GH and CM accessed and verified underlying data reported in the manuscript; all authors reviewed and agreed with the final version of the manuscript to submit for publication.

## Data sharing statement

De-identified data from the trial which is not found in this manuscript can be made available upon request from a qualified investigator.

## Declaration of interests

AL, BB, CSM and HK are shareholders of SYMBYX Pty Ltd; All other authors declare no competing interests.

## Appendix A Supplementary data

Supplementary Materials Table S1

Protocol Parkinsons trial Sydney with remote contingency
