# Supplementary figures and images for: A novel transcranial photobiomodulation device to address motor signs of Parkinson's disease: a parallel randomised feasibility study
Source: eClinicalMedicine. 2023 Dec 1;66:102338. doi: 10.1016/j.eclinm.2023.102338 (PMC10716000; doi:10.1016/j.eclinm.2023.102338)

Suppl Materials


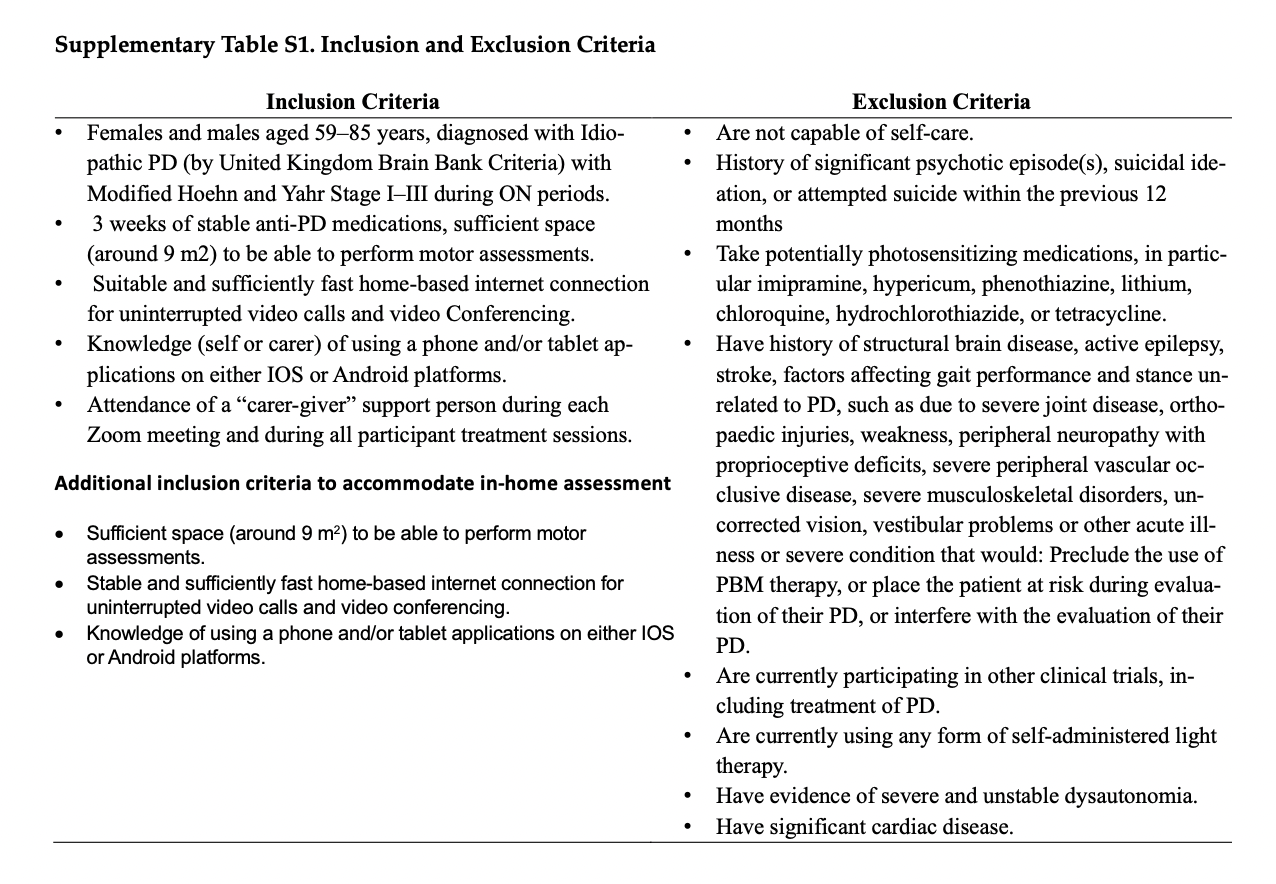

Supplement: Supplementary Materials Table S1 [file mmc1.docx]
