# Supplementary material for: A novel transcranial photobiomodulation device to address motor signs of Parkinson's disease: a parallel randomised feasibility study
Source: eClinicalMedicine. 2023 Dec 1;66:102338. doi: 10.1016/j.eclinm.2023.102338 (PMC10716000; doi:10.1016/j.eclinm.2023.102338)
Supplement: Protocol Parkinsons trial Sydney with remote contingency [file mmc2.docx]

**Evaluation of dose of Photobiomodulation (Light) Therapy and Physiotherapy for improving quality of life outcomes and mobility in Parkinson’s Disease (Sydney)**

**STUDY INVESTIGATOR(S):**

Co-ordinating Principal Investigator (CPI)

Dr Ann Liebert - Honorary Director of Photomolecular Research, ARI, San Hospital;

Adjunct Senior Lecturer, Dept Medicine, University of Sydney

[ann.liebert@outlook.com](mailto:ann.liebert@outlook.com)

Chief Investigators (CI):

Prof Liisa Laakso - Senior Research Fellow, Mater Hospital, Brisbane

Conjoint Lecturer, University of Queensland, Brisbane

[liisa.laakso@mater.uq.edu.au](mailto:liisa.laakso@mater.uq.edu.au)

Prof Hosen Kiat - Professor of Cardiology, Macquarie University, Marsfield (PhD Student supervisor)

[hosen.kiat@chi.org.au](mailto:hosen.kiat@chi.org.au)

Dr Brian Bicknell - Honorary Fellow, Faculty of Health Science, Australian Catholic University

[brian.bicknell@acu.edu.au](mailto:brian.bicknell@acu.edu.au)

Associated Investigators (AI):

Dr Roberta Chow - Brain and Mind Centre, Sydney University, Camperdown

Photobiomodulation Clinic, Specialist and GP Centre, San Hospital, Wahroonga

[robertachow102@gmail.com](mailto:robertachow102@gmail.com)

Dr Greg Bennett - Specialist in Gerontology, San Hospital

[gregorybbennett@gmail.com](mailto:gregorybbennett@gmail.com)

Dr Sandra Elias - Medical Registrar, Royal Prince Alfred Hospital, Camperdown

sandraelias342@gmail.com

Dr Delena Caagbay- Physiotherapist, Artarmon Physiotherapy, Artarmon

[delena.caagbay@gmail.com](mailto:delena.caagbay@gmail.com)

PhD Student:

Ms Claire McGee – Torrens University Australia, Sydney
[Claire.mcgee@Torrens.edu.au](mailto:Claire.mcgee@Torrens.edu.au)

**1. INTRODUCTION**

Parkinson’s disease (PD) is the second most common neurodegenerative disease, which affects at least 70,000 Australians and is increasing in occurrence with an aging population. Currently there is no cure and very few options to treat symptoms, with the exception of levodopa whose effects are temporary. It is proposed in this trial to test the effectiveness of photobiomodulation treatment (PBMt) to alleviate some of the symptoms of PD and improve quality of life. PBMt entails the delivery of non-thermal light (either laser or LED) as a therapy. PBMt has a proven track record to reduce pain, accelerate healing and reduce inflammation. More recently PBMt has undergone trials to assess its affect in the treatment of stroke, depression, traumatic brain injury and Alzheimer’s disease. The proposed Phase 1 trial follows on from two other completed trials using PBMt to treat the symptoms of PD; one in Brisbane and one in Adelaide.

**2. BACKGROUND**

Parkinson’s Disease (PD) is the second most common neurological disease and refers to a progressive neurodegenerative disorder (i.e., degeneration of the nervous system). Clinical manifestations in people with PD are altered muscle tone, bradykinesia (slowness of movement), choreatic movement (constant writhing), rigidity (increased muscle tone during movement), akinesia (difficult in initiating and stopping movement) and resting tremor (shaking movements). The prevalence of PD in Australia is approximately 70,000 with 32 new cases diagnosed every day resulting in a very substantial total economic cost to the Australian community of $9.9b per annum (Deloitte Access Economics, 2015).

PD presents as a result of nerve cell loss in an area of the midbrain (the substantia nigra) (Calabresi et al, 2015). In humans, it is estimated that when motor (movement) signs first appear and a diagnosis is possible, there is already a 27% loss of dopamine nerve cells.

The cause of PD is unclear. Some evidence suggests that it may be caused by exposure to a neurotoxin or defective genes. Another hypothesis is associated with dysfunction of the powerhouse of the cell, the mitochondria. It is within the mitochondria that cellular energy is created by the production of adenosine triphosphate (ATP). ATP is the fuel for all cellular (including nerve) function. Under certain conditions there can be a progressive accumulation of mutations in mitochondrial DNA that reduces mitochondrial efficiency and ATP yield. This process leads to an increase in toxic elements (reactive oxygen species), generating oxidative stress and subsequent nerve degeneration (Calabresi et al, 2013; Fuhrer et al, 2014).

The current medical treatment for PD is dopamine replacement drug therapy. In some cases, more radical management, such as implantation of deep brain stimulation (DBS) can be offered (Calabresi et al., 2013). Dopamine drug therapy (DDT) aims to replace the dopamine lost from the system, while DBS aims to introduce stimulation to basal ganglia pathways impaired by the loss of the dopamine.

DDT, although effective in assisting communication between cells in the impaired mid brain (Calabresi et al., 2015) has many side effects, (more common in the medium term) that may cause alterations in other neural pathways (Ferrazzoli 2016). Identifying alternative non-invasive methods of stimulating cellular activity in neurones within the impaired mid-brain may prove highly beneficial to people with PD.

Physiotherapy has been established as an effective and complementary treatment for the management of PD regardless of medical interventions (Tomlinson et al., 2013). The aim of physiotherapy is to offer strategies and improve strength and capacity for some of the impairments such as gait disturbances, reduced strength, coordination and balance responses, assist in movement initiation strategies and maintain dexterity and fine motor control. Current physiotherapy, therefore, does much to improve the quality of life and functional capacity, however, it is unable to halt disease progression.

Photobiomodulation therapy (PBMt; also known as light therapy, low intensity laser therapy or low-level laser therapy) has been used in humans for a wide range of conditions such as musculoskeletal disorders, inflammatory conditions and neurogenesis. PBMt is a safe, non-invasive, and non-thermal modality that is based on a substantial body of research dating back to the 1960s (Chung, 2012). The mechanisms of action are proposed to involve the stimulation of mitochondria by the absorption of photons in cytochrome c oxidase, resulting in increased ATP production, leading to reduced oxidative stress, anti-inflammatory effects, improved cellular energy, increased synthesis of enzymes, and increased focal cerebral blood flow (Hamblin, 2016). Most encouragingly, recent research has reported neuroprotection against brain cell degeneration, stimulated by PBMt in animal models of PD (Darlot et al, 2016; Johnstone et al 2014, Johnstone et al, 2015).

To date, the basic science research, and anecdotal clinical evidence offer support for the benefits of PBMt in PD (Shaw et al 2012; Johnstone et al 2014, Johnstone et al, 2015; Darlot et al, 2016). In a monkey model in which a Parkinson’s Disease state was induced by a neurotoxin, the application of PBMt reduced the signs of motor dysfunction as well as promoted neural protective responses and in some cases neuro-regeneration of impaired nerve cells (Darlot et al, 2016). Further research, reports PBMt is well tolerated and may be valuable in improving sleep-wake cycles in patients with PD (Videnovic et al., 2017). PBMt has also been shown to be safe in other brain-related conditions. Studies have applied both transcranial (e.g., WARP-LED) or intracranial (e.g., optical fiber device) methods to deliver light therapy at power intensities ranging from 1–700 mW/cm^2^ and have reported no adverse effects on brain tissue structures and function (Moro et al, 2014; Hamblin, 2016; Saltmarche et al, 2017).

In the helmet experiment (Stone and Mitrofanis, 2012) one of the researchers in our team (JM) analysed the survival of dopaminergic neurones in the mouse brain (substantia nigra pars compacta) and showed that body-only PBMt exposure is effective in protecting dopaminergic centres of the brain, although less protective than head-and-body radiation. The protective effect of PBM may therefore be mediated partially by a systemic or indirect effect, involving quite different mechanisms to those involved in the transcranial effect (as being measured in the Brisbane arm of the study). The site of PBMt application need to be assessed to understand where it may be best in future to apply the therapy to optimise treatment.

The applicability of PBMt, the positive animal study responses and the lack of side effects suggest that PBMt could provide a viable adjunct to current treatments for PD. No study has yet explored this method of treatment in humans. We propose to trial the PBMt in a series of case studies to best identify the type of application, the site of application and dose rate that may produce positive changes in clinical signs and functional capacity of people with PD. Given that abscopal effects (where treatment in one part of the body can influence remote sites) are known for PBMt, our research seeks to understand whether site of PBMt application influences clinical signs and functional capacity of people with PD, in the same way that dosing may do. Hence, a Brisbane arm of our parallel studies (with separate HREC application) will evaluate the effect of PBMt to the head of eligible participants with PD whilst the Sydney arm will evaluate the effect of PBMt on the abdomen and spine. The abdomen is a critical site due to recent evidence proposing a link between the gut and brain associated with PD (Pellegrini et al, 2015). The spine as part of the central nervous system is a key site of application due to its links with the brain. This HREC application is specific to the Sydney arm of our parallel studies.

Investigators in our team have already established the efficacy and safety of PBMt in animals (rodents and primates) using an invasive technique (Darlot et al, 2016). Based on recent studies of transcranial PBMt in humans with traumatic brain injury, stroke and dementia, we know that it is not necessary to apply PBMt utilizing a surgical method. We intend to use a non-invasive remote site approach in order to measure any abscopal effect.

The Sydney proof-of-concept trial follows on from proof-of-concept trials which have been completed in Brisbane and Adelaide. The Brisbane trial was a randomised placebo trial using transcranial photobiomodulation and showed significant differences between the treated and placebo group. The Adelaide trial was a waiting-room design with transcranial, intranasal, cervical spine and abdominal photobiomodulation; results are still being analysed but look very encouraging, including potentially positive changes in the microbiome. The Sydney trial will be a case-matched trial and use abdominal and cervical spine photobiomodulation only. The Sydney trial will be conducted over a 12-week treatment period and will assess a number of outcome measures, including motor abilities, cognitive function, microbiome changes, cytokine levels and potential Parkinson’s disease marker changes, as well as self and carer assessments of wellness. The trial is to be conducted in two stages: an initial cohort will be assessed in September, to begin treatment in October. A further cohort will begin treatment in August 2021. The funding for the Sydney trial has been provided by the San Foundation and from private donors. Additional funding ($27,000) has been obtained to conduct the second stage of the trial. The results from all three trials will be used to argue for a large-scale, randomised, double-blinded trial.

The idea for this study emerged after a previous study evaluating the application of photobiomodulation in participants with PD was completed (approved by Griffith University HREC). Ethics approval for this study is being sought through the AHCL HREC. This study will be a simplified blinded placebo/sham-controlled trial with the following sites:

- Northshore Musculoskeletal and Laser Physiotherapy Clinic, Suite 6, 110-114 Hampden Rd, Artarmon, NSW.
- Photobiomodulation Clinic, Specialist and GP Centre, San Hospital, 185 Fox Valley Rd, Wahroonga, NSW
- Suite 211, the Tulloch building, San Hospital, 185 Fox Valley Rd, Wahroonga, NSW

The sponsor and the supplier of devices for this study will be SYMBYX Biome Pty Ltd, 2/50 Yeo Street, Neutral Bay, NSW 2089

**3. AIM OF STUDY**

The main aim of the trial is to intervene in with photobiomodulation, delivered via LED, to determine the efficacy of using this modality to treat the symptoms of Parkinson’s disease and determine if symptoms of Parkinson’s disease are able to be modified to improve quality of life outcomes.

**4. OBJECTIVES**

1. to determine the effect of PBMt over a 12-week period on a variety of fine and course motor symptoms
2. to determine the effect of PBMt over a 12-week period, on a cognitive function, including social cognition
3. to determine the effect of PBMt over a 12-week period, on the composition of the intestinal and oral microbiome

**5. HYPOTHESIS**

5a. Primary Hypothesis: PBMt will have no adverse effects on PD participants

5b. Secondary Hypothesis: PBMt will reduce the symptoms of PD

5c. Tertiary Hypotheses: PBMt will alter the microbiome

**6. STUDY DESIGN**

Stage 2

Blinded, placebo/sham-controlled trial consisting of 40 participants. Group 1 (20 participants) will be

participants with Parkinson’s disease receiving treatment and Group 2 (20 participants) will be a placebo/sham-control group receiving no treatment. All groups will begin intervention in

August 2021. Treatment will consist of PBMt to the head and nose (either active or

placebo treatment), 3 times per week for 12 weeks. Outcome measures to be collected by Specialist

Examiners and the Physiotherapists.

**7. STUDY SETTING/LOCATION**

- Northshore Musculoskeletal and Laser Physiotherapy Clinic, Suite 6, 110-114 Hampden Rd, Artarmon, NSW.
- Photobiomodulation Clinic, Specialist and GP Centre, San Hospital, 185 Fox Valley Rd, Wahroonga, NSW
- Suite 211, the Tulloch building, San Hospital, 185 Fox Valley Rd, Wahroonga, NSW

**8. STUDY POPULATION**

Two groups of participants will be recruited: 20 participants with PD receiving active intervention (Group 1) and 20 participants with PD receiving sham/placebo intervention (Group 2)

**8a. Inclusion criteria**

- Females and males aged 60–85 years
- Diagnosed with Idiopathic PD (by UK Brain Bank Criteria) with Modified Hoehn & Yahr (H&Y) Stage I-III during ON periods
- ≥3weeks of stable anti-Parkinson’s Disease medication.

**8b. Exclusion** **criteria**

Patients will be excluded from the study, if they:

- Are not capable of self-care
- Have a cognitive impairment with Montreal Cognitive Assessment (MOCA) score of <24
- History of significant psychotic episode(s) within the previous 12 months
- History of suicidal ideation or attempted suicide within previous 12 months.
- Take potentially photosensitizing medication, especially imipramine, hypericum, phenothiazine, lithium, chloroquine, hydrochlorothiazide, or tetracycline
- have a history of structural brain disease, active epilepsy, stroke or acute illness, factors affecting gait performance and stance such as severe joint disease, orthopaedic injuries, weakness, peripheral neuropathy with proprioceptive deficits, severe peripheral vascular occlusive disease, severe musculoskeletal disorders, uncorrected vision, vestibular problems or other severe conditions that would:
  - preclude the use of PBM therapy
  - place the patient at risk during evaluation of their PD, or
  - interfere with the evaluation of their PD
- have cardiac disease
- Patients who are currently participating in other trials regarding the treatment of PD, such as advanced therapies (Duodopa, Apomorphine, DBS).

**8c. Sample size**

- Group 1 - 20 participants
- Group 2 - 20 participants

**9. STUDY COHORT**

- Groups 1 participants with PD receiving active intervention.
- Groups 2 participants with PD receiving sham/placebo intervention.

**10. RECRUITMENT STRATEGY**

Potential participants will be recruited from specialised private clinics and neurologists at the Sydney Adventist Hospital through paper advertisements and referrals from specialist clinicians.

**11. STUDY OUTCOMES**

**10a. Primary Outcome:** Improved quality of life

**10b. Secondary Outcomes:**

- Course motor control (up-and-go test; timed walk; step test)
- Fine motor control (spiral test; 9-hole peg test; writing task)
- Balance (static; tandem)
- Cognition (Montreal Cognitive Assessment)
- Self-assessment (PDQ39 questionnaire; PDSS sleep scale)
- Carer diary of daily living
- Smell identification test
- Microbiome analysis

**12. STUDY PROCEDURES**

**11a. Study procedure**

The PBMt intervention will consist of 3 treatment sessions per week for 12 weeks (Groups 1). Participants in the active treatment group will receive transcranial light treatment with a PDNeuro LED helmet device (parameters given in Appendix 1). The treatment areas are 20 points (

154.5 joules total energy). The treatment time is 24 minutes. Participants in the placebo/sham group (Group 2) will receive the same apparent treatment as Group 1, except that they will be “treated” with sham transcranial LED devices that delivers no light.

**Outcome** **measures**

Group 1 and 2: Outcome measures will be performed at the commencement of the trial before intervention (baseline, week-0), after 4 weeks of treatment, and at the end of the trial (12 weeks of treatment), and at 6-months (3-months of zero intervention wash out). In addition to the physiognomy measurements (weight, girth), motor coordination, balance, cognition, social cognition, smell test, carer diary and self-assessment outcome measures, participants will also be asked to provide faecal and saliva samples (for microbiome analysis) on 2 occasions; 3 samples before treatment begins and 3 samples at the end of the treatment period (12 weeks).

**12b. Measurement tools used**

- Demographic data
- Physiognomic measurement (height and weight)
- Step test (Appendix 2)
- 10 meter walk test (Appendix 2)
- Spiral test (Appendix 2)
- Montreal Cognitive Assessment (MoCA) test (Appendix 3)
- Timed up-and-go (TUG) (Appendix 2)
- 9-hole peg board (Appendix 2)
- Static balance (feet apart, feet together, eyes open, eyes closed) (Appendix 2)
- Tandem balance (eyes open, eyes closed) (Appendix 2)
- Hand-writing assessment
- PDQ-39 questionnaire (Appendix 4)
- Parkinson’s disease sleep scale (PDSS) questionnaire (Appendix 5)
- Social cognition questionnaires (Appendix 6)
- Carer diary of daily activities (Appendix 7)
- Microbiome analysis (faecal and saliva sample)
- Heart rate variability
- Smell Identification test

**12c. Safety considerations/Patient safety**

PBMt is considered a safe treatment. In over 50 years of research on the effects of PBM, there have been no published results of harm when used within the correct dose window. Dosing protocols for the PD trial have been based on both clinical experience by members of the research team (LL, AL) and others (Videnovic et al., 2017), as well as results of PBMt studies in primates and mice (Darlot et al, 2016). No side-effects of PBMt or safety concerns using PBMt, have been reported in either of the two previous arms of the PD trial performed in Brisbane and Adelaide.

**13. DATA ANALYSIS**

**13a. Outcome measurements**

Baseline data for all outcome measures collected before the beginning of treatment will form the reference point for analyses.

**13b. Statistical analysis**

As a series of N=1 studies, it is anticipated that only basic statistical analysis will be performed. For each outcome measure, descriptive data (mean, standard deviation) will be calculated. From this data, “minimally important difference” (MID) scores will be computed based on ½ SD of each measure. This is a common MID measure (Norman et al, 2003) based on the distribution of the participant scores at baseline and provides a sensitive indicator of significant change over time for N=1 case studies. As such, it does not suffer from a lack of statistical power that would be evident with more traditional ANOVA approaches with small sample sizes. The number of participants showing improvement (i.e., difference between two time points > MID) can be compared between time points with chi-square analyses.

Participants showing improvement in the Sydney arm of the multi-centre Parkinson’s trial will be compared to the Brisbane and Adelaide arms (chi-square analysis) in order to establish optimum PBM areas of treatment and doses regimens.

**14. ETHICAL CONSIDERATIONS (including consent process)**

Screening for eligible participation in the trial will take place by phone if a patient contacts the trial site for further information and before written consent. Further screening may be necessary at the first appointment. Prior to a patient’s arrival at the clinic, standardised forms are sent to patients for completion in preparation for the initial physiotherapy assessment. For the purposes of this study, in addition to these forms, two Participant Information Sheets will be sent to the patient; one for them and one for their nominated carer (e.g., spouse). This will inform the potential participants of the Parkinson’s Disease Research project at North Shore Physiotherapy and invite them to read the enclosed information sheet. If patients wish to learn more information, the information sheet will direct them to contact the administrative team at North Shore Physiotherapy and the SAN hospital prior to their appointment. Should a patient indicate an interest in participating, the Principal Coordinating Investigator will contact the patient by phone, answer any questions and further explain the project and determine eligibility to participate in the project. Patients with Parkinson’s Disease as well as their nominated carer will be asked to re-read the Participant Information Sheet and bring the consent form for signing to their first appointment.

A potential participant will have time to consider participation in the trial between receipt of the Participant Information Sheet, the telephone discussion with the Principal Coordinating Investigator and the first appointment. The length of this period can be up to four weeks before an appointment time becomes available.

Participants in Group 2 (sham/placebo treatment) will be offered active light treatment with the active light devices at the conclusion of the 12-week study free of charge.

**14a. Maintenance of records, retention and storage of data**

Only the investigators named on the front of the participant information and consent document will have access to the research records, other than the participants.

Participants with PD will have access to their own data and to diary data only and on request. Participant carers will only have access to their diary data on request. There will be no cross-over of access by participants to any other data.

The research records will be stored in a secure place without reference to participant names. They will be coded to de-identify participants, and these codes will be used throughout the analysis of the results to ensure that the researchers are the only people who could match results with participant names. The data will be stored on an excel spreadsheet with a passcode to the data set, with a copy on an external hard drive. Only the CPI and CIs on this project will have access to the excel data sheet. Once the study is complete, all information that could be identifiable will be destroyed, and only the coded (de-identified) data will be kept for the period required by the HREC. The information and consent forms will be stored in a locked filing cabinet at Griffith University for 15 years.

**15. OUTCOMES AND SIGNIFICANCE**

The results of this study (will inform the researchers as to which dose and site of application, if any, influences outcome measures for people with Parkinson’s Disease. This information will provide the foundation dose methodology for a larger placebo-controlled trial for this patient population as well as information required to adequately power a larger trial.

**16. Anticipated Publication/Presentation**

De-identified results will be presented at conferences and in publications. It is anticipated that at least 4 publications can be produced from the data generated from the PD trials.

**17. CONTINGENCY PLAN**

In the event that the currently proposed protocol is unable to proceed due to uncontrollable and/or unforeseen restrictions or regulations (such as those imposed by government or global bodies e.g., COVID-19 pandemic lockdown restrictions) that directly or indirectly affect:

1. The study site(s).
2. The participant’s ability to participate in the currently proposed study protocol.
3. The researcher’s ability to conduct the currently proposed study protocol.

Several changes constituting a new contingency protocol will be formulated and implemented to adhere to such restrictions/regulations (See Appendix 8.). A layman’s version of the contingency protocol will also be included as part of the PICF and handed to participants as part of the recruitment and consent process, including a separate clause to indicate that participants have received, read, and understood the contingency protocol. Information about recruitment for the study will be advertised on public media platforms, with contact details available for interested potential participants to contact the research team directly.

**18. REFERENCES**

Calabresi P, Castrioto A, Di Filippo M, et al. New experimental and clinical links between the hippocampus and the dopaminergic system in Parkinson’s disease. *Lancet Neurol*. 2013;12(8):811-821.

Calabresi P, Ghiglieri V, Mazzocchetti P, et al. Levodopa-induced plasticity: a double-edged sword in Parkinson’s disease? *Philos Trans R Soc B-Biological Sci*. 2015;370(1672):1-14.

Chung H, Dai T, Sharma SK, et al. The nuts and bolts of Low-level Laser (Light) Therapy. *Ann Biomed Eng*. 2012;40(2):516-533.

Darlot F, Moro C, El Massri N, et al. Near-infrared light is neuroprotective in a monkey model of Parkinson’s disease. *Ann Neurol*. 2016;79(1):59-75.

Deloitte Access Economics 2015 *Living with Parkinson's Disease: An updated economic analysis 2014*. Accessed at: http://www.parkinsons.org.au/deloitte-report_copy.

Ferrazzoli D, Carter A, Ustun FS, et al. Dopamine replacement therapy, learning and reward prediction in Parkinson’s Disease: Implications for rehabilitation. *Front Behav Neurosci*. 2016;10:121.

Fuhrer H, Kupsch A, Hälbig TD, et al. Levodopa inhibits habit-learning in Parkinson’s disease. *J Neural Transm*. 2014;121(2):147-151.

Hamblin, M. R. Shining light on the head: photobiomodulation for brain disorders. BBA Clinical 6(2016): 113-124.

Johnstone DM, el Massri N, Moro C, et al. Indirect application of near infrared light induces neuroprotection in a mouse model of parkinsonism – An abscopal neuroprotective effect. *Neuroscience*. 2014;274:93-101.

Johnstone D M, Moro C, Stone J, et al. Turning on lights to stop neurodegeneration: The potential of near infrared light therapy in Alzheimer's and Parkinson's Disease. *Front Neurosci*, 2015,9:500.

Moro C, Massri N El, Torres N, et al. Photobiomodulation inside the brain: a novel method of applying near-infrared light intracranially and its impact on dopaminergic cell survival in MPTP-treated mice. *J Neurosurg*. 2014;120(3):670-683.

Norman GR, Sloan JA, Wyrwich KW. Interpretation of changes in health-related quality of life: the remarkable universality of half a standard deviation. Med Care. 2003; 41 (5): 582 – 592.

Pellegrini C, Antonioli L, Colucci R, et al Gastric motor dysfunctions in Parkinson’s disease: Current pre-clinical evidence. *Parkinsonism Rel Disord*. 2015; 21 (12):1407-1414.

Saltmarche AE, Naeser MA, Ho KF, et al. Significant improvement in cognition in mild to moderately severe dementia cases treated with transcranial plus intranasal photobiomodulation: Case series report. *Photomed Laser Surg*. 2017;10.

Sampson TR, Debelius JW, Thron T, Janssen S, Shastri GG, Ilhan ZE, et al. Gut microbiota regulate motor deficits and neuroinflammation in a model of Parkinson’s disease. *Cell*. 2016;167:1469-80. e12.

Scheperjans F, Aho V, Pereira PAB, Koskinen K, Paulin L, Pekkonen E, et al. Gut microbiota are related to Parkinson's disease and clinical phenotype. *Movement Disorders.* 2015;30:350-8.

Shaw V E, Peoples C, Spana S, et al. Patterns of cell activity in the subthalamic region associated with the neuroprotective action of Near-Infrared light treatment in MPTP-treated mice. *Parkinson’s Dis*, 2012;296875.

Stone J, Mitrofanis J The helmet experiment: an observation of the mechanism of action of LED-sourced infrared light. World Association for Laser Therapy WALT2012 biennial congress, Gold Coast, September 27-30.

Tomlinson CL, Patel S, Meek C, et al. Physiotherapy versus placebo or no intervention in Parkinson’s disease. *Cochrane Database Syst Rev*. 2013;(9):CD002817.

Videnovic A, Klerman EB, Wang W, et al. Timed light therapy for sleep and daytime sleepiness associated with Parkinson’s Disease. *JAMA Neurol.* 2017;74(4):411.

Zesiewicz TA, Baker MJ, Wahba M, Hauser RA. Autonomic nervous system dysfunction in Parkinson’s disease. *Current Treatment Options in Neurology.* 2003;5:149-60.

**Appendix 1**

PDNeuro (LED) Primary Specifications:

- PDNeuro Helmet LED
- LED positions: Transcranial - 20 LED locations, each containing 1 x infra-red (IR) 810nm LED and 1x red 660nm LED; nasal – 1 x IR 810nm LED.
- Average power output: 660nm LEDs 71.2 mW; 810nm transcranial LEDs 103.4 mW; 810nm nasal LED 20mW.
- Duty cycle: 50% all diodes
- Treatment time: 660nm LEDs 12 minutes; 810nm transcranial LEDs 12 minutes; 810nm nasal LED 24 minutes.
- Total light energy delivered: 660nm LEDs 51.3 joules; 810nm transcranial LEDs 74.4 joules; 810nm nasal LED 28.8 joules; TOTAL = 154.5
- pulse frequency: all diodes 40Hz.

**Appendix 2**

**9-hole peg board test:**

Instructions and demo video for this can be found at:

<https://www.physio-pedia.com/Nine-Hole_Peg_Test>

**Step Test:**

Participants will place their foot up and back down on a standard height step (10cm) as many times as possible in 30 seconds. Both legs will be tested.

**TUG (timed up-and-go):**

Participants will be instructed to stand up from a chair, walk 3 meters (previously marked on the floor), turn around, return and sit down again. A standard chair 45 cm in height with arm supports at a height of 65 cm (using the floor as a reference) will be used. At the verbal command “Are you ready? Go” the participant will start the test. Time will be counted from the moment when the volunteer’s trunk no longer touches the back of the chair, to the point when the volunteer’s trunk returns to leaning against the back of the same chair. Time in seconds will be used for analysis. The TUG test will be performed three times: once normally, once carrying a cup of water (motor TUG) and once counting backwards in 2 from 100 (cognitive TUG). Instructions and video demo at:

<https://www.physio-pedia.com/Timed_Up_and_Go_Test_(TUG)>

**10m walk test:**

Participants will be instructed to walk at a pace closest to their habitual gait speed over a 10 meter distance, with time measured to travel the middle 8 meters to account for acceleration and deceleration. The variables of gait cycle that will be analyzed are: gait speed (m/s), cadence (steps/min), stride time (s), step length (cm), base of support (cm), single support (% of the gait cycle), and double support (% of the gait cycle).

Instructions and demo video at:

<https://www.physio-pedia.com/10_Metre_Walk_Test>

**Static and dynamic balance:**

The Mini Balance Evaluation Systems Test (Mini-BESTest) is a 14-item clinical test used to measure four components of balance control: anticipatory postural adjustments (sit to stand, rise to toes, stand on 1 leg), postural responses (stepping in four different directions), sensory orientation (stance – eyes open; foam surface – eyes closed; incline – eyes closed) and dynamic balance during gait (gait during change speed, head turns, pivot turns, obstacles; cognitive “get up and go” with dual task). Each item is scored from 0 (unable or requiring help) to 2 (normal), and the maximum score is 28 points.

**Spiral drawing test**

This is a pen-and-paper test. Publication embedded:

**Appendix 3**

**
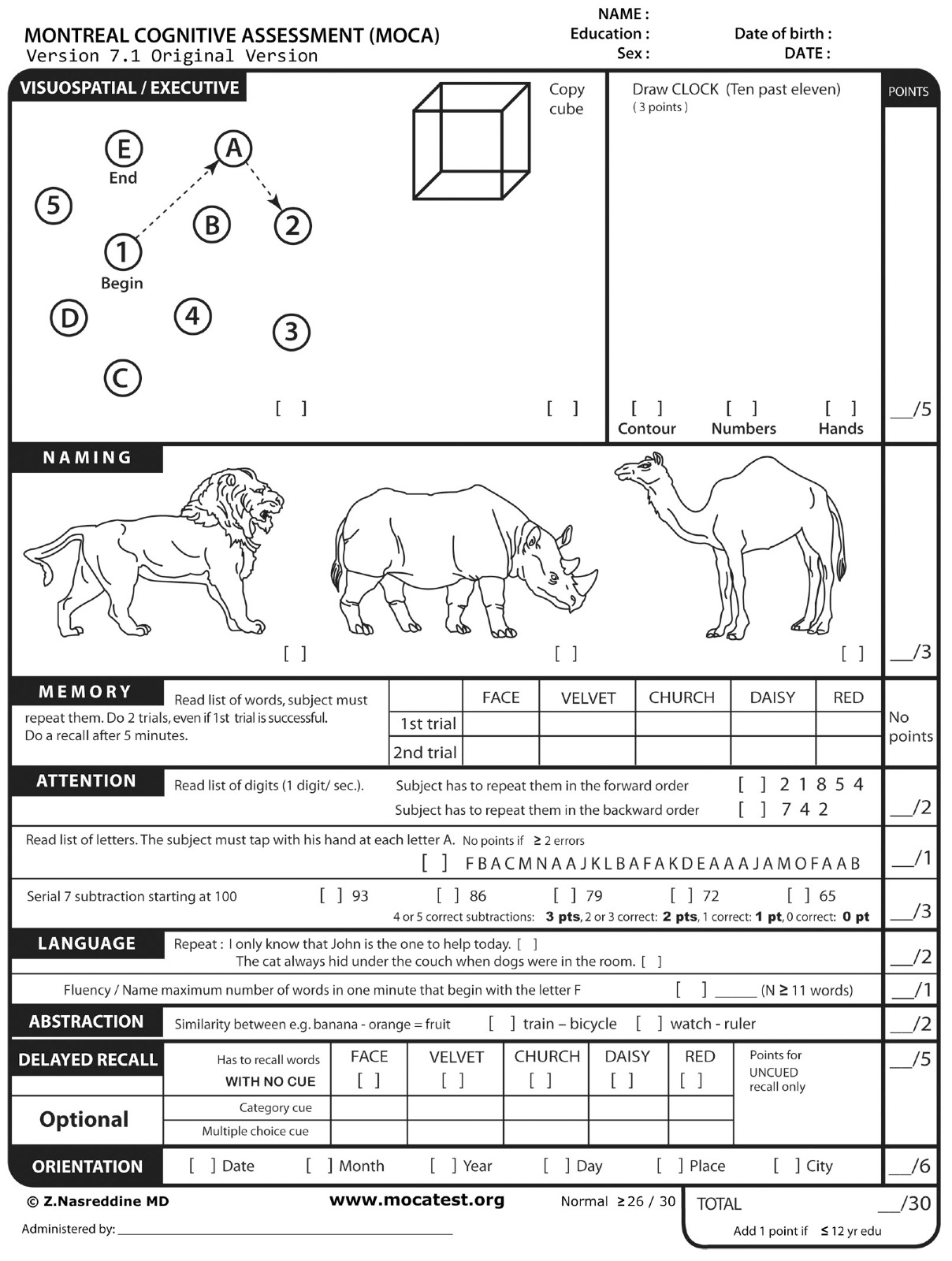
**

**Appendix 4**

**Appendix 5**

**Appendix 6**


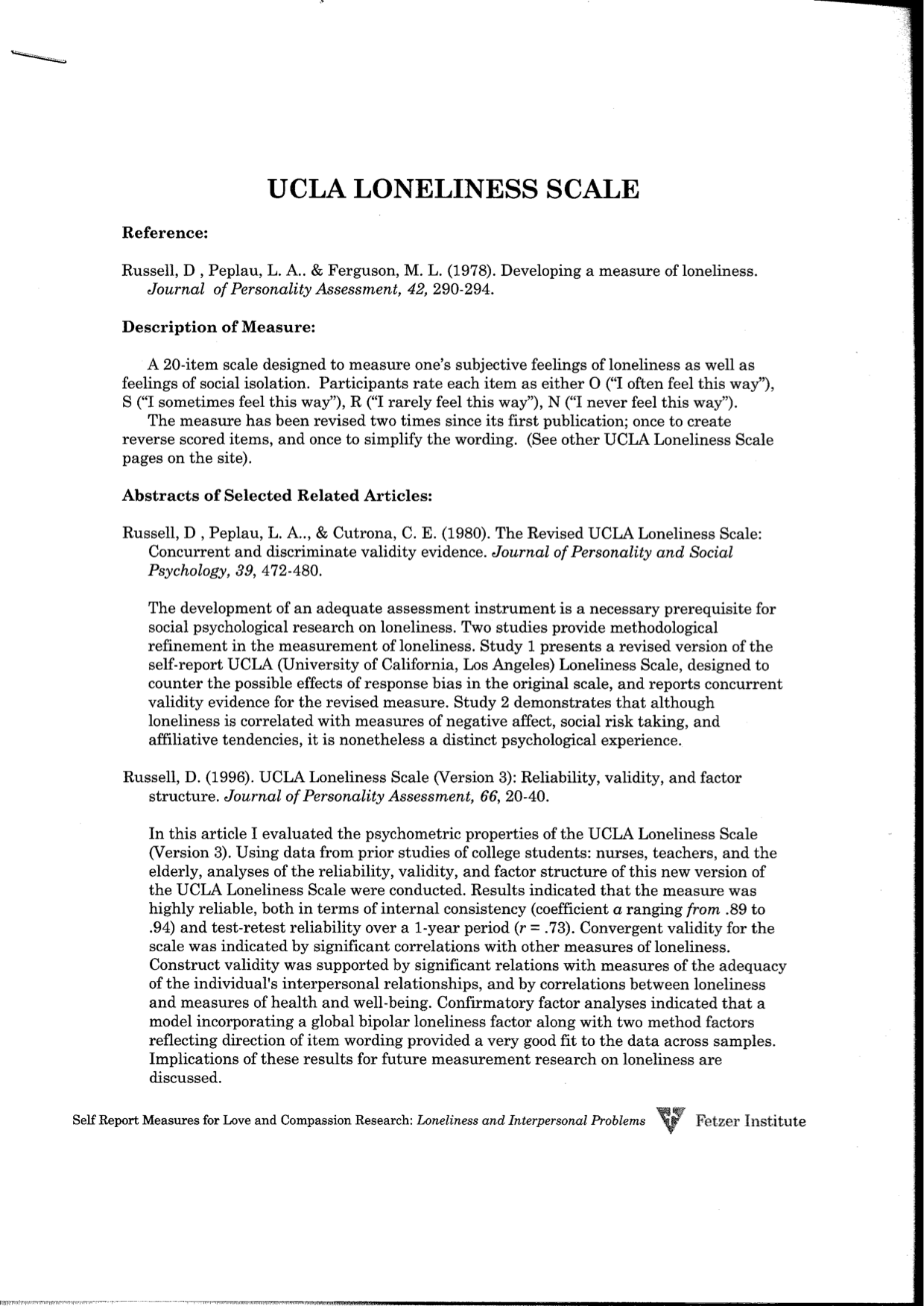


**Appendix 7**


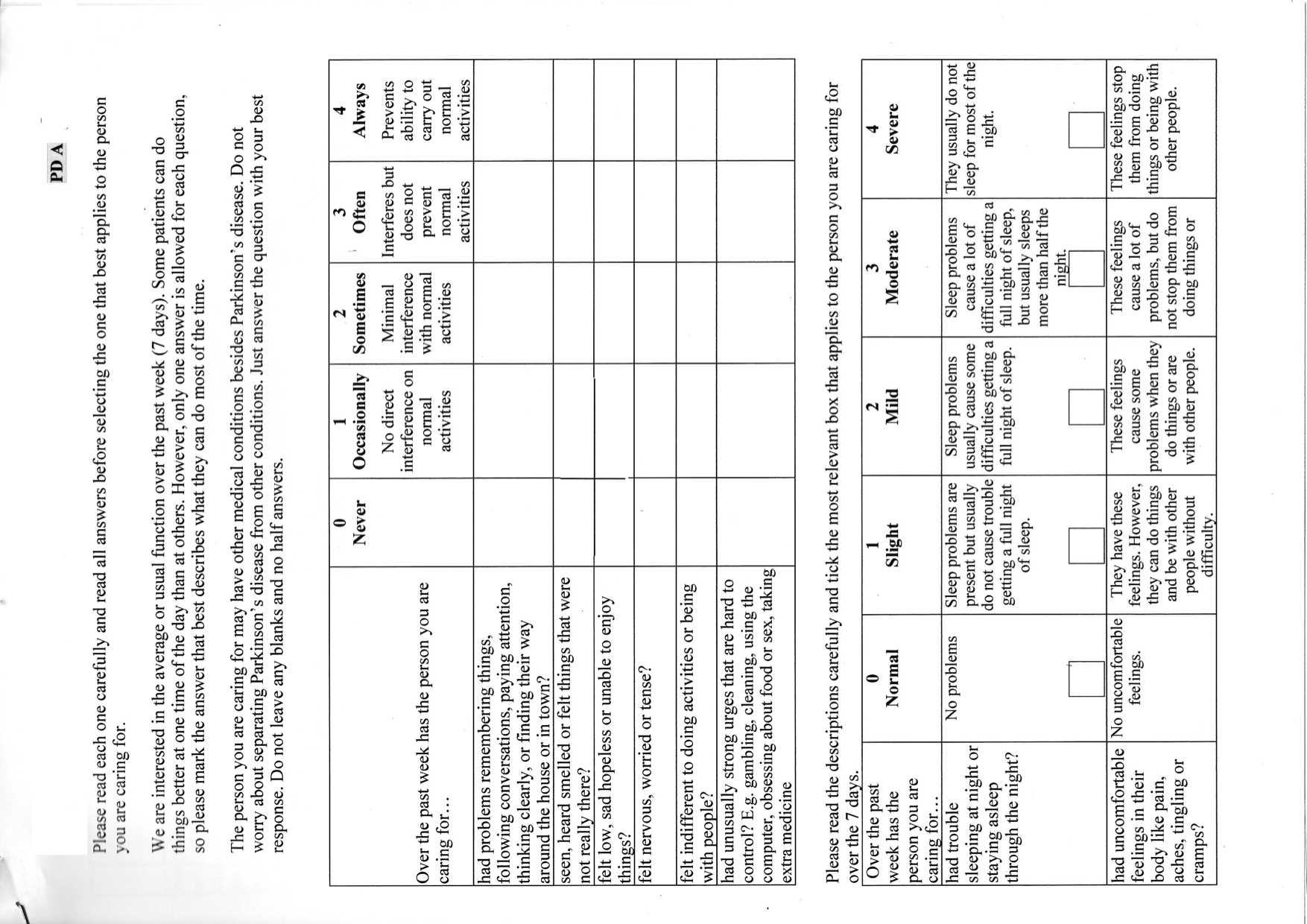


**Appendix 8**

Contingency protocol

In the event that the currently proposed protocol is unable to proceed due to uncontrollable and/or unforeseen restrictions or regulations, the following changes will be implemented to the existing protocol constituting a new contingency protocol.

1. **Study design**
   1. Treatment will consist of home-based (in the participant’s own home) PBMt to the head and nose (either active or placebo treatment), 6 daily treatments per week for 12 weeks. Outcome measures to be supervised and data collected via the use of video link by Specialist Examiners and the Physiotherapists.
2. **Study setting/location**
3. Study location for both treatment outcome measures assessments will be changed to the participant’s own home.
4. **Inclusion criteria**
5. In addition to the inclusion criteria already stipulated, eligible participants will require:

   - Sufficient space (around 9 m^2^) to be able to perform motor assessments.
6. - Stable and sufficiently fast home-based internet connection for uninterrupted video calls and video conferencing.

   - Knowledge (self or carer) of using a phone and/or tablet applications on either IOS or Android platforms.**Study procedure**
7. In the absence of researcher/specialist-directed intervention, to ensure blinding of participants from group allocations and to reduce the change of participants inadvertently realising that they are in the sham, participants will be informed that there are 3 groups rather than 2 (Group 1: receiving red and infra-red light; Group 2: receiving infra-red light only; Group 3: receiving sham). In fact, there will remain to be 2 groups (Group 1: receiving red and infra-red light; Group 2: receiving sham).
8. **Outcome measures**
9. Outcome measures will be performed at the commencement of the trial before intervention (baseline, week-0), then after 2, 4, 6, 8, 10-weeks of treatment, and at the end of the trial (12-weeks of treatment), and at 6-months (3-months of zero intervention wash out).
10. All outcome measures will be conducted and recorded using a combination of self-reported assessment, visual assessment via video link with a specialist examiner (during live video or pre-recording by the participant).

1. **Consent process**
2. All previous in-person appointments during the consent process will now be conducted via video-link. For example:

   - Should a patient indicate an interest in participating in the study, the Principal Coordinating Investigator will contact the patient by phone or video link to answer any questions or to further explain the project and determine eligibility to participate in the project.

   - Patients and nominated carers will then be asked to re-read the Information Sheet and deliver the consent form electronically for live countersigning during their first video-link appointment.
3. **Additional screening, selection and other considerations**
4. In addition to the assessment of inclusion criteria and screening protocols being conducted via video telehealth communication, potential risk factors including falls risk, and cardiac comorbidities will be assessed live by qualified nominated geriatrician and cardiologist.
5. Additional exclusion criteria will include evidence of severe and unstable dysautonomia, recent cardiac surgeries (in the past 3-months), unstable arrhythmias (for a duration of 3-months), evidence of cardiac dysautonomia.
6. As an added duty of care, extra time will be taken to ensure that the overall speed and pace of any conversations between the research team and participant during screening and outcome measures assessments are suitable to account for the absence of in-clinic and personal supervision.
7. In addition to the original recruitment strategy, we will also employ public media platforms to present information regarding our study.
